# Postnatal Care Service Utilization and Associated Factors among Women Who Gave Birth in the Last 12 Months prior to the Study in Debre Markos Town, Northwestern Ethiopia: A Community-Based Cross-Sectional Study

**DOI:** 10.1155/2016/7095352

**Published:** 2016-06-28

**Authors:** Miteku Andualem Limenih, Zerfu Mulaw Endale, Berihun Assefa Dachew

**Affiliations:** ^1^College of Medicine and Health Science, Department of Midwifery, University of Gondar, P.O. Box 196, Gondar, Ethiopia; ^2^College of Medicine and Health Science, Institute of Public Health, Department of Epidemiology and Biostatistics, University of Gondar, P.O. Box 196, Gondar, Ethiopia

## Abstract

Improving maternal and newborn health through proper postnatal care services under the care of skilled health personnel is the key strategy to reduce maternal and neonatal mortality. However, there were limited evidences on utilization of postnatal care services in Ethiopia. A community based cross-sectional study was conducted in Debremarkos town, Northwest Ethiopia. Cluster sampling technique was used to select 588 study participants. Bivariate and multivariable logistic regression model was fitted to identify factors associated with postnatal care utilization. Odds ratio with 95% confidence interval was computed to determine the level of significance. Postnatal care service utilization was found to be 33.5%. Awareness about maternal complication (AOR: 2.72, 95% CI (1.71, 4.34)), place of delivery of last child (AOR: 1.68, 95% CI: (1.01, 2.79)), outcome of birth (AOR: 2.71, 95% CI (1.19, 6.19)), delivery by cesarean section (AOR: 4.82, 95% CI (1.86, 12.54)), and delivery complication that occurred during birth (AOR: 2.58, 95% CI (1.56, 4.28)) were factors associated with postnatal care service utilization. Postnatal care service utilization was found to be low. Increasing awareness about postnatal care, preventing maternal and neonatal complication, and scheduling mothers based on the national postnatal care follow-up protocol would increase postnatal care service utilization.

## 1. Introduction

Postnatal care (PNC) is the care provided to women and newborn in the first six weeks after birth [1]. Postnatal period is a time interval that starts from the birth of the baby to six consecutive weeks with the recommended time of visit, that is, 6–24 hours, 3–6 days, and 6 weeks following childbirth [1]. Even though the health of mothers is mostly regarded as the health of the society, an estimated 287 000 maternal deaths occurred worldwide [2]. Maternal mortality remains unacceptably high across much of the developing world especially Sub-Saharan Africa (SSA) and South Asia accounting for 87% of maternal deaths [3].

Every year, three million neonates die within their first month of life, representing nearly 40% of all deaths of children under the age of five, and almost all newborn deaths are in developing countries [3]. In Sub-Saharan Africa, one in nine children dies before the age of five, which is more than 16 times higher than the average number of deaths in developed regions [4].

The fact that 18 million women in Africa currently do not give birth in a health facility poses challenges for planning and implementing postnatal care for women and their newborns [5]. In Africa, most mothers and newborns did not visit the health institution following birth, indicating that postnatal care programs are among the weakest of all reproductive and child health programs [6].

A large proportion of maternal and neonatal deaths occur during 48 hours following childbirth. These first two days following birth are critical time to prevent complications arising from the childbirth. As a result, PNC services help to safeguard women from complications following birth and provide important opportunity to assess the infant development to offer newborn care [7].

Postnatal care coverage is extremely low in Ethiopia. More than nine in ten mothers received no postnatal care at all and only 7% received postnatal care within the first two days after the birth. In the Amhara region, where the study is conducted, percentage of women with postnatal checkup in the first two days after they gave birth was only 5.1% [8]. Due to this fact, maternal mortality and morbidity level in Ethiopia are still the highest in the world, 676 deaths per 100,000 live births [9].

A study done in Jabitena district, Ethiopia, on factors affecting utilization of postnatal care services showed that only 20.2% delivering mothers got PNC services [10]. Another cross-sectional study done on knowledge, perception, and utilization of postnatal care services in Gondar Zuria district, Ethiopia, showed that utilization of postnatal care service among mothers who gave birth in the last one year was 66.8% [11].

Researches indicate that PNC services utilization is affected by several factors including maternal age, educational level of the women, occupational status of women and husbands, place of delivery, mode of delivery, number of pregnancies, awareness about obstetric related danger sign, and awareness about PNC services [10, 12–14]. However, the determinants of utilization of PNC services are not the same across different cultures and socioeconomic status within a society. Thus, assessing factors affecting utilization of postnatal care service in different setup area is very important to improve maternal and child health services. Therefore, this study aimed to assess postnatal care service utilization and associated factors among women who gave birth in the last 12 months prior to the study in Debre Markos town, Northwestern Ethiopia. The finding of this study will help to improve postnatal care service utilization in the region.

## 2. Methods 

### 2.1. Study Setup

A community-based cross-sectional study was conducted from 1st to 30th November, 2014, at Debre Markos town. The town is located 300 KM to the Northwest of Addis Ababa, the capital city of Ethiopia. According to the 2013 population projection estimate, there were 101,582 residents and more than half of them were females. There are 4 health centers, 17 clinics, 7 health posts, and 1 referral hospital providing postnatal care service in the town.

### 2.2. Sample Size Calculation and Sampling Procedure

**Figure 1:**
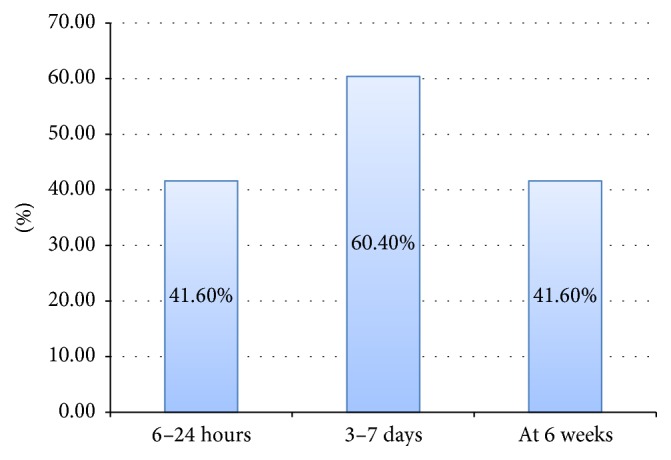
Postnatal care services on recommended time of visit among postnatal care service utilizers at Debre Markos town, Northwest Ethiopia, December, 2014 (*n* = 197).

The single population proportion formula was used to calculate the sample size considering the following assumptions: proportion of women using postnatal care services 78.3% [12], 95% confidence level, 5% margin of error (absolute level of precision), and design effect of two in order to account for intercluster variability. In the recruitment of the study participants, the present study has undertaken cluster sampling technique. With this regard, minimum sample size required becomes 527. For possible nonresponse during the survey, the final sample size increased by 10%. Thus, including 10% nonresponse rate, the final sample size becomes 580. A lottery method was employed to select four of the seven cluster kebeles. Finally, all eligible mothers in the selected clusters were included in the study. This made the final number of respondents 588 (Figure 1).

### 2.3. Data Collection and Analysis

Data were collected using a structured and pretested questionnaire via face-to-face interview at the participant's home. The questionnaire was first prepared in English and then translated into local language (Amharic) and back to English to ensure consistency. Four diploma midwives and one BSC midwife supervisor were involved in the data collection process. One-day training was given to the data collectors and the supervisor.

Women who gave birth one year prior to the study period and who lived in the study area for at least six months were included in this study. Postnatal care service utilization was measured as the use of postnatal care services by mothers following childbirth till 42 days at least once after home birth and, for those mothers who gave birth at the health institution, mothers who came back for postnatal care services at least once after they were discharged to their homes.

Data were entered using EPI-INFO version 3.5.3 and exported to SPSS version 20 for further analysis. Descriptive statistics were carried out to characterize the study population using different variables. Both bivariate and multiple logistic regressions were used to identify associated factors. Variables having *p* value ≤ 0.2 in the bivariate analyses were fitted into a multiple logistic regression model to control the effects of confounding. Crude and adjusted odds ratio with their 95% CI were calculated to determine the strength and presence of association. *p* value of 0.05 was considered to declare the level of significance.

### 2.4. Ethical Considerations

Ethical clearance was obtained from the ethical review committee of Department of Midwifery, University of Gondar. An official letter of cooperation was written to the Debre Markos town administration. After explaining the purpose of the study, written informed consent was obtained from each of the study participants. Participants were also informed that participation was on a voluntary basis and that they can withdraw at any time if they are not comfortable about the questionnaire. Personal identifiers were not included in the written questionnaires to ensure participants' confidentiality.

## 3. Results 

### 3.1. Sociodemographic Characteristics of Respondents

A total of 588 mothers who gave birth in the last 12 months were interviewed. The mean age of the respondents was 28.54 years with SD of ±4.6 years. Sixty percent of the mothers were in the age range of 20–29 years. Regarding the marital status of the respondents, the majority, 494 (84%), of them were married. Five hundred fifty-five (94.4%) of the respondents belong to Amhara by ethnicity.

Nearly one-fifth of the respondents, 107 (18.2%), were unable to read and write and 138 (23.5%) of them attend secondary education and above. Concerning their husbands' educational status, 288 (49%) attended secondary education and above, and 193 (32.8%) of respondents husbands were farmers by occupation. Regarding average monthly income, 226 (38.4%) of the mothers have monthly income less than 25 dollars. From total respondents, 192 (32.7%) mothers got information about postnatal care services from health personnel (Table 1).

### 3.2. Women Awareness about the Postnatal Care Related Services

From a total of 588 respondents, 238 (40.5%) mothers were aware of PNC services. Regarding the awareness about complications that can occur during postpartum period, 226 (38.4%) mothers were aware of maternal complications and 219 (37.2%) were aware of neonatal complication.

### 3.3. Obstetric Characteristics of Respondents

 Among respondents, three hundred five (51.9%) mothers were categorized as para two to para four followed by para one, 168 (28.6%). Among the respondents, 50 (8.5%) mothers faced stillbirth, while they gave last birth. Three hundred thirty-six (57.1%) mothers gave their last birth at health institution. As to the mode of delivery, most respondents (452 (76.9%)) delivered by spontaneous vaginal delivery.

Of all respondents, nearly half, 301 (51.2%), of the mothers had antenatal care follow-up during recent pregnancy. Regarding obstetric complication during the last pregnancy, less than one-fourth, 118 (20.1%), of the mothers experienced at least one complication. Two hundred seventeen (36.9%) mothers faced delivery complication while they gave a recent birth (Table 2).

### 3.4. Utilization of Postnatal Care Services among Mothers

From a total of respondents, one hundred ninety-seven (33.5%) mothers were utilized in postnatal care services. Of these, thirty-two (16.2%) mothers got the service three times and above and most, 113 (57.4%), of postnatal care services utilizers got the service by urban health extension workers and only 7.1% of utilizers got the service by physicians.

Among mothers who got postnatal care services, 119 (60.4%) of the respondents stated that they got postnatal care services within 3–7 days following birth (Figure 1).

### 3.5. Reasons for Nonutilization of PNC Services

**Figure 2:**
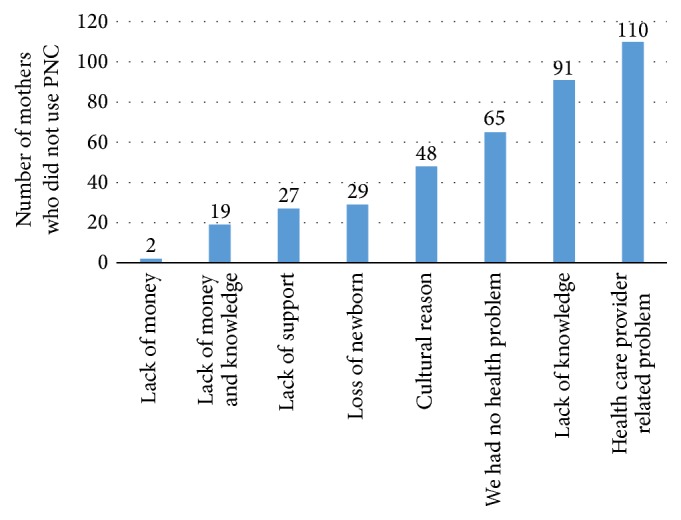
Reasons for not attending postnatal care services given by the study participants at Debre Markos town, Northwest Ethiopia, December, 2014 (*n* = 391).

Different reason was given by the mothers for not attending postnatal care services (*n* = 391). Out of these, the most frequent reason was that the health personnel did not schedule them to return for postnatal care services within 42 days following birth, followed by a lack of knowledge, 91 (23.3%), about the advantage and availability of postnatal care (Figure 2).

### 3.6. Factors Associated with Postnatal Care Service Utilization

Awareness about maternal complication (AOR: 2.72, 95% CI: 1.71, 4.34), place of delivery (AOR: 1.68, 95% CI: 1.01, 2.79), outcome of birth (AOR: 2.71, 95% CI: 1.19, 6.19), mode of delivery (AOR: 4.82, 95% CI: 1.86, 12.54), and delivery complication (AOR: 2.58, 95% CI: 1.56, 4.28) were found to be significantly associated with postnatal care service utilization in multivariate logistic regression analysis.

Those mothers who were aware of maternal complications that can occur during postpartum period were 2.7 times more likely to use postnatal care services than mothers who were not aware of maternal complications that can occur during postpartum period (AOR: 2.72, 95% CI: 1.71, 4.34).

Moreover, mothers who gave their latest child at health institution were 1.68 times (AOR: 1.68, 95% CI: 1.01, 2.79) more likely to get postnatal care service utilization when compared with those mothers who gave birth at home.

Mothers who gave birth to a live neonate were 2.7 times more likely to get postnatal care services than mothers who gave stillbirth (AOR: 2.71, 95% CI: 1.19, 6.19).

Furthermore, mothers who gave birth by cesarean section were 4.8 times more likely to get postnatal care services than mothers who gave birth by spontaneous vaginal delivery (AOR: 4.82, 95% CI: 1.86, 12.54).

Those mothers who faced birth related complication while giving birth were 2.58 times more likely to get postnatal care services utilization than mothers who did not face complication while giving birth (AOR: 2.58, 95% CI: 1.56, 4.28) (Table 3).

## 4. Discussion

This study revealed that the proportion of women who got postnatal care services was 33.5%. This finding was very low as compared with the study done in Adwa town, North Ethiopia (78.3%) [12]. This difference may be because those mothers who lived in Adwa were more aware of the advantage of PNC follow-up (89.9%) than mothers in this study area (40.5%). It may also be due to sample size difference [12].

However, this finding is much higher than the national and Amhara regional report which was stated as follows: the proportion of postnatal care service utilization was 7% and 5.1%, respectively [9]. This difference may be due to difference in study setting, in which EDHS study included both rural and urban residents while this study was done only in urban mothers. Thus, mothers who reside in urban area may have good awareness about the advantage of postnatal care service and had better educational status than the rural residents. In addition, mothers in the town may get easy access to health institution and health care providers when compared with rural residents.

Moreover, this finding is higher than that of the finding of the study done in West Bank, Palestine (19%) [15], and in Jabitena district, Amhara region, Ethiopia (20.2%) [10]. Methodological and time difference may explain this variation.

Postnatal care services utilization in this study was lower than the study done on antenatal and postnatal care service utilization in Southern Ethiopia [13] and the study done in Gondar Zuria district, Ethiopia [11]. This discrepancy may be because mothers of this study area are less educated and less aware of the importance of postnatal care service utilization and it might be also due to methodology difference.

Mothers who were aware of maternal complications that can occur during postnatal period were 2.7 times more likely to use postnatal care services than mothers who were not aware (AOR: 2.72, 95% CI: 1.71, 4.34). This finding is consistent with the study done in Royal Government of Cambodia which revealed that mothers who were aware of maternal complications during postpartum period were 1.63 times more likely to use postnatal care services than those who were not aware [16]. The possible explanation to this might be due to the fact that awareness of maternal complication is an important factor in motivating women and their families to attend health care service at the earliest opportunity with the intention of prevention and early detection, to be managed if any sign of complication occurred, have better decision for health, and have good attitude to go to health facility. Furthermore, women's educational level and being exposed to mass media have influence on awareness and have implication on utilization of postnatal care services [10, 17].

Place of delivery was one of the strongest predictors of postnatal care service utilization. Those mothers who gave birth to their latest child at health institution were 1.68 times (AOR: 1.68, 95% CI: 1.01, 2.79) more likely to get postnatal care service utilization when compared with those mothers who gave birth to their latest child at home. This finding is in line with EDHS report, 2011 [9], a study done in Jabitena district Amhara region, Ethiopia [10], Gondar Zuria district, Ethiopia [11], Royal king of Cambodia [16], Bangladesh [18], and Nepal [19], which indicates that giving birth at health institution has significantly associated with postnatal care service utilization. The possible explanation for this strong positive association of PNC services utilization with place of delivery can be attributed to the fact that women who gave their last birth in health institution have greater opportunity for health education related to PNC services at the time of delivery and thus get access to learning about the types, benefits, and availabilities of PNC services during their stay in the health institutions. This exposure increases health care seeking behavior to prevent maternal and neonatal complications compared to those mothers who gave birth at home. Furthermore, those women who gave birth at home belong to more traditional cohort and thus become less likely to use postnatal care services [12].

The result of this study revealed that the use of postnatal care services has been significantly influenced by delivery complications while giving birth. Mothers who faced delivery complication while giving birth were 2.58 times more likely to get postnatal care services than mothers who did not face complication while giving birth (AOR: 2.58, 95% CI: 1.56, 4.28). This is in line with the study done in Palestine [15]. This might be because those mothers who faced complication while giving birth were given special emphasis by health personnel regarding health education and could be scheduled more seriously for postnatal care follow-up. Furthermore it might be due to exposure of complication increase fear of additional health complication and increase interest in check up.

Outcome of birth was another variable which has significant association with postnatal care service utilization. Mothers who gave birth to live neonate were 2.7 times more likely to get postnatal care services than mothers who gave stillbirth (AOR: 2.71, 95% CI: 1.19, 6.19). This may be due to the fact that good outcome of birth has positive implication on postnatal care follow-up for mothers as well as for newborns.

Utilization of postnatal care services was significantly influenced by mode of delivery. Mothers who delivered by cesarean section were 4.8 times more likely to get postnatal care services than mothers who delivered by spontaneous vaginal delivery (AOR: 4.82, 95% CI: 1.86, 12.54). This is in line with study done in maternal health care service utilization in rural Ethiopia [20]. This might be due to fear of complication and exposure to health care services, to prevent further complication and increase health care seeking behavior. In addition to this, the health personnel put complicated mothers while giving birth in a special corner of care to prevent incredible complications and they want to check mothers weather they had improved or not; this makes the mothers go to health institutions.

Sixty-six point five percent of mothers did not attend postnatal care services. The most frequent reason was health care worker related problem followed by lack of knowledge, 91 (23.3%), about the advantage of postnatal care services and they did not face problems regarding both herself after delivery and her neonate. This study agrees with the study done in Adwa [12].

### 4.1. Limitation of the Study

Despite the fact that those mothers who gave birth in the last 12 months are included in the study, there might be a recall bias. The cross-sectional nature of the study does not confirm the definitive cause and effect relationship.

## 5. Conclusion

The proportion of postnatal care services utilization in the last 12 months was found to be low. Awareness about maternal complication, outcome of birth, mode of delivery, place of delivery, and ever faced delivery complication while giving last birth were factors significantly associated with postnatal care service utilization. The health care providers and policy makers are recommended to increase the awareness mothers on postnatal care services, to prevent maternal and neonatal complication and to schedule mothers based on the national postnatal care follow-up protocol in order to increase postnatal care service utilization.

## Figures and Tables

**Table 1:** Sociodemographic characteristics of the study participants at Debremarkos town, December 2014 (*n* = 588).

Variables	Frequency (%)
*Age of the mother at interval (mean, SD: 28.5 ± 4.6)*	
<20	21 (3.6%)
20–29	353 (60%)
30–39	190 (32.3%)
40–49	24 (4.1%)
*Marital status of the mother*	
Married	494 (84%)
Divorced	35 (6%)
Widowed	10 (1.7%)
Single	36 (6.1%)
Separated due to work	13 (2.2%)
*Religion of the mother*	
Orthodox	509 (86.6%)
Muslim	63 (10.7%)
Protestant	16 (2.7%)
*Ethnicity *	
Amhara	555 (94.4%)
Oromo	15 (2.6%)
Tigre	18 (3.1%)
*Educational status of the mothers*	
Cannot read and write	107 (18.2%)
Can read and write	220 (37.4%)
Elementary education (1–8)	123 (20.9%)
Secondary education and above	138 (23.5%)
*Occupational status of the mother*	
Merchant	91 (15.5%)
Housewife	220 (38.8%)
Farming	44 (7.5%)
Government employee	136 (23.1%)
Daily laborer	89 (15.1%)
*Educational status of the husband (n* = 507)	
Cannot read and write	45 (8.9%)
Can read and write	80 (15.8%)
Elementary education (1–8)	94 (18.5%)
Secondary education and above	288 (56.8%)
*Husband occupational status (n* = 507)	
Merchant	185 (36.5%)
Farming	67 (13.2%)
Government employee	193 (38.1%)
Daily laborer	62 (12.2%)
*Average monthly income*	
<25$	226 (38.4%)
25$–75$	186 (31.6%)
>75$	176 (29.9%)
*Source of information about PNC services*	
From health personnel	192 (32.7%)
From peers or neighbors	189 (32.1%)
From radio or television	207 (35.2%)

**Table 2:** Obstetric characteristics of respondents who gave birth in the last 12 months in Debremarkos town, northwest Ethiopia, December, 2014 (*n* = 588).

Variables	Frequency (%)
*Parity*	
One	168 (28.6%)
Two–four	305 (51.8%)
Five and above	115 (19.6%)
*Outcome of birth*	
Alive	538 (91.5%)
Stillbirth	50 (8.5%)
*Place of delivery *	
Home	252 (42.9%)
Health institution	336 (57.1%)
*Mode of delivery *	
Spontaneous vaginal delivery	452 (76.9%)
Instrumental delivery	105 (17.9%)
Cesarean section	31 (5.2%)
*ANC visit during last pregnancy*	
Yes	301 (51.2%)
No	287 (48.8%)
*Obstetric complication during last pregnancy *	
Yes	118 (20.1%)
No	470 (79.9%)
*Delivery complication during last birth *	
Yes	217 (36.9%)
No	371 (63.1%)

**Table 3:** Bivariate and multivariable analysis of factors associated with PNC utilization among the study participants at Debremarkos town, December, 2014 (*n* = 588).

Variables	PNC utilization		COR (95% CI)	AOR (95% CI)
	Yes	No		
*Educational status of mothers*				
Cannot read and write	38	69	1	1
Can read and write	54	166	0.59 (0.36, 0.98)	1.16 (0.64, 2.08)
Elementary education (1–8)	37	86	0.78 (0.46, 1.24)	1.05 (0.59, 1.87)
Secondary education and above	68	70	1.76 (1.05, 2.96)	1.44 (0.79, 2.60)
*Average monthly income*				
<25$	67	159	1	1
25$–75$	52	134	0.96 (0.60, 1.41)	1.38 (0.83, 2.31)
>75$	78	98	1.89 (1.25, 2.85)	1.15 (0.65, 2.04)
*Source of information about PNC*				
From health personnel	84	108	1.95 (1.92, 2.96)	1.22 (0.75, 1.98)
From peers/neighbors	54	135	1.00 (0.64, 1.58)	0.71 (0.42, 1.21)
From radio/television	59	148	1	1
*Awareness about PNC services*				
Yes	122	116	3.89 (2.96, 5.53)	0.69 (0.35, 1.40)
No	75	275	1	1
*Awareness about maternal complication *				
Yes	124	102	4.81 (3.34, 6.95)	2.72 (1.71, 4.34)^*∗*^
No	73	289	1	1
*Awareness about neonatal complication*				
Yes	115	104	3.87 (2.69, 5.56)	0.79 (0.36, 1.79)
No	82	287	1	1
*Place of delivery *				
Home	45	207	1	1
Health institution	152	184	3.80 (2.58, 5.59)	1.68 (1.01, 2.79)^*∗*^
*Outcome of birth *				
Alive	188	350	2.45 (1.16, 5.14)	2.71 (1.19, 6.19)^*∗*^
Stillbirth	9	41	1	1
*Mode of delivery *				
Spontaneous vaginal delivery	134	318	1	1
Instrumental	39	66	1.40 (0.46, 2.11)	1.12 (0.82, 5.51)
Cesarean section	24	7	8.14 (3.42, 19.34)	4.82 (1.86, 12.54)^*∗*^
*ANC follow-up for last pregnancy *				
Yes	138	163	3.27 (2.27, 4.71)	1.01 (0.54, 1.91)
No	59	228	1	1
*Obstetric complication during last pregnancy *				
Yes	62	56	2.75 (1.82, 4.15)	1.23 (0.73, 2.09)
No	135	335	1	1
*Delivery complication during recent birth *				
Yes	112	105	3.59 (2.50, 5.14)	2.58 (1.56, 4.28)^*∗*^
No	85	286	1	1

^**∗**^Significantly associated with *p* value < 0.05.
